# High Resolution Analysis of Respiratory Syncytial Virus Infection In Vivo

**DOI:** 10.3390/v11100926

**Published:** 2019-10-10

**Authors:** Waleed Aljabr, Stuart Armstrong, Natasha Y. Rickett, Georgios Pollakis, Olivier Touzelet, Elaine Cloutman-Green, David A. Matthews, Julian A. Hiscox

**Affiliations:** 1 King Fahad Medical City, Research Center, 59046 Riyadh 11525, Saudi Arabia; 2 Institute of Infection and Global Health, University of Liverpool, Liverpool L3 5RF, UK; sarmstro@liverpool.ac.uk (S.A.); N.Rickett@liverpool.ac.uk (N.Y.R.); g.pollakis@liverpool.ac.uk (G.P.); julian.hiscox@liverpool.ac.uk (J.A.H.); 3 National Institute of Health Research, Health Protection Research Unit in Emerging and Zoonotic Infections, Liverpool L3 5RF, UK; 4 School of Medicine, Dentistry & Biomedical Sciences, Queen’s University Belfast, Belfast BT9 7BL, UK; O.Touzelet@qub.ac.uk; 5 Great Ormond Street Hospital, London WC1N 3JH, UK; elaine.cloutman-green@gosh.nhs.uk; 6 School of Medical Sciences, University of Bristol, Bristol BS8 1TD, UK; d.a.matthews@bristol.ac.uk

**Keywords:** respiratory syncytial virus, proteomics, RNAseq, nasopharyngeal aspirate, host response, quasispecies, clinical sample, respiratory disease

## Abstract

Human respiratory syncytial virus (HRSV) is a major cause of pediatric infection and also causes disease in the elderly and those with underlying respiratory problems. There is no vaccine for HRSV and anti-viral therapeutics are not broadly applicable. To investigate the effect of HRSV biology in children, nasopharyngeal aspirates were taken from children with different viral loads and a combined high throughput RNAseq and label free quantitative proteomics approach was used to characterize the nucleic acid and proteins in these samples. HRSV proteins were identified in the nasopharyngeal aspirates from infected children, and their abundance correlated with viral load (Ct value), confirming HRSV infection. Analysis of the HRSV genome indicated that the children were infected with sub-group A virus and that minor variants in nucleotide frequency occurred in discrete clusters along the HRSV genome, and within a patient clustered distinctly within the glycoprotein gene. Data from the samples were binned into four groups; no-HRSV infection (control), high viral load (Ct < 20), medium viral load (Ct = 20–25), and low viral load (Ct > 25). Cellular proteins associated with the anti-viral response (e.g., ISG15) were identified in the nasopharyngeal aspirates and their abundance was correlated with viral load. These combined approaches have not been used before to study HRSV biology in vivo and can be readily applied to the study the variation of virus host interactions.

## 1. Introduction

Human respiratory syncytial virus (HRSV) is one of the major lower respiratory tract pathogens in infants with consistent annual outbreaks. 90% of infants are infected with HRSV at least once within the first two years of life [1,2]. Elderly patients [2] and patients with chronic heart and lung conditions such as asthma and immunocompromised patients are also at risk of severe infection and subsequent disease [3,4,5,6]. Co-infection with respiratory pathogens may exacerbate clinical symptoms [7]. According to the World Health Organization, there are approximately 60 million people infected with HRSV and 160,000 associated deaths every year [8]. HRSV exists as two distinct lineages known as subgroups A and B, which represent two lines of divergent evolution with both genetic and serologic differences. While both subgroup A and B viruses contribute to overall HRSV disease, some studies have suggested that subgroup A isolates may have slightly different clinical presentations [9] and effects on host cell signaling [10] compared to subgroup B viruses. In contrast, other studies have found no difference in the severity of illness caused by either subgroup [11]. However, a subgroup A clade virus was shown to be associated with more severe illness than other subgroup A clades [11].

HRSV belongs to the genus *Pneumovirus* of the family *Paramyxoviridae* and the order *Mononegavirales* [12,13]. The viral genome consists of a non-segmented ∼15-kb RNA of negative polarity that encodes 11 proteins. As with all the members of the *Mononegavirales*, the genomic RNA of HRSV is always tightly bound by the viral nucleoprotein (N) and maintained as a helical N-RNA ribonucleoprotein (RNP) complex [14]. The RNP is used a template for transcription and replication of viral mRNA and genomic RNA by the RNA-dependent RNA polymerase (RdRp) complex, consisting of large polymerase protein (L) and its cofactor phosphoprotein (P). While N, P, and L are sufficient to mediate viral replication, transcription activity also requires the M2-1 protein, which functions as a polymerase processivity cofactor [15]. A feature of the *Mononegavirales* is that the RdRp is present in the virus particle to initiate viral mRNA transcription upon release of the RNP in the cytoplasm. The order of genes along the genome for HRSV from 3′ to 5′ is non-structural proteins 1 and 2 (NS1 and NS2), N, P, matrix (M), small hydrophobic (SH), glycoprotein (G), fusion (F), M2, and L. Synthesis of HRSV mRNAs has been proposed to follow a transcription gradient, with genes at the 3′ end of the genome (e.g., NS1 and NS2) being transcribed more than genes at the 5′ end of the genome (e.g., the L gene), which thus provides a control of viral protein abundance.

Macrophages and epithelial cells are involved in the innate immune response to HRSV. Many cytokines and chemokines including IL-8/ CXCL8, IP-10/CXCL10, MCP-1/CCL2, MIP-1, CCL3, MIP- 1b/CCL4, RANTES/CCL5, IL-6, TNF-, IL-1, and IFN are produced by these cell types in response to HRSV infection, and can be detected in enhanced amounts in respiratory secretions of children, hospitalized for HRSV infection [16]. During HRSV infection of cells, anti-viral signaling pathways are activated (e.g., references [17,18,19]), and this can depend on the genotype of the virus [10,20]. However, HRSV proteins can also modulate the host response to infection. NS1 and NS2 proteins inhibit IFN regulatory factor 3 (IRF3) activation and this modulates type I IFN induced signaling and has other effects on host cell biology [21]. HRSV also down regulates the production and function of IFN [22] by inhibiting toll-like receptor (TLR) signaling and mitochondrial antiviral signaling protein [23]. Moreover, a reduced apoptosis and enhanced survival of the infected cells results from activation of the phosphoinositidide 3-kinase pathway by NS1 and NS2 [24]. Soluble factors are released into the supernatant of infected cells [25]. During HRSV infection there is an initial strong neutrophil response (with associated inflammation) followed by T-cell lymphopenia and a pulmonary CD8^+^ T-cell response [19]. A protective antibody response is associated with B-cell stimulating factors [19]. Immunity to reinfection is not complete and this may, in part, be due to the ability of the virus to interfere with antibody induced neutralization [26].

Whilst many studies have focused on the interaction of HRSV with the host cell, we wanted to use high resolution approaches to characterize HRSV infection in vivo. Given that HRSV is predominantly a childhood infection, we wanted to explore the effect of the virus on this population. Ethically and practically it is difficult to take lung biopsies from this patient group, particularly from a control group who have no obvious signs of illness. Instead, diagnostic left over nasopharyngeal aspirates were used as a proxy for high resolution approaches to identify molecular changes in the respiratory tract as a result of virus infection. Two high resolution approaches were used: quantitative proteomics using liquid chromatography with tandem mass spectrometry (LC-MS/MS) and RNA sequencing (RNAseq) on an Illumina 2500. These techniques provided information on both viral and cellular proteins and mRNAs and how these varied between HRSV-infected and uninfected children.

## 2. Materials and Methods 

### 2.1. Ethics Statement and Source of Nasopharyngeal Aspirates

Nasopharyngeal aspirates were collected from HRSV sub-group A positive children (<1–9 years-old, median age = 2.5, IQR = 2.5) admitted to Great Ormond Street Hospital for Children in London, UK. HRSV diagnosis was confirmed by the Film Array multiplex polymerase chain reaction (PCR) system respiratory panel (Biomerieux diagnostics). This panel detects the 20 most common respiratory pathogens; adenovirus, coronavirus HKU1, coronavirus NL63, coronavirus OC43, human metapneumovirus, human rhinovirus/Enterovirus, influenza A, influenza A H1, influenza A H1-2009, influenza A/H3, influenza B, parainfluenza virus 1, parainfluenza virus 2, parainfluenza virus 3, parainfluenza virus 4, HRSV, Bordetella pertussis, Chlamydophila pneumoniae, and Mycoplasma pneumoniae. For this study the nasopharyngeal aspirates represented discarded NHS left-over samples and there was no identifying information. Nasopharyngeal aspirate samples from HRSV-negative children of similar age (<1–9 years-old, median age = 2, IQR = 2.25), obtained during the same HRSV season, were used as controls, and no pathogens were detected in this group using the Film Array. The nasopharyngeal aspirate sampling was carried out as per the hospital’s guidelines. Briefly, a catheter was inserted into the nose, directed posteriorly and towards the opening of the external ear, in order to reach the posterior pharynx. Suction was applied and the catheter was slowly withdrawn using a rotating movement, remaining less than 10 s in the nasopharynx. The catheter was then rinsed with a small volume of sterile 0.9% saline solution to ensure adequate specimen volume. Samples were stored at −80 °C until use.

### 2.2. Viruses and Cells

HEp-2 cells were kindly provided by Ralph Tripp (University of Georgia) and grown at 37 °C with 5% CO2 in Dulbecco’s modified Eagle’s medium (DMEM) high glucose (4.5 g/L) (Life Technologies, Grand Island, New York, USA) supplemented with 5% (v/v) FBS and penicillin (100 IU/mL) and streptomycin (100 µg/mL) (Pen Strep, Life Technologies, Renfrew, UK). Commercially available primary pediatric bronchial epithelial cells (donors aged 2–9 years) (Lonza, Basel, Switzerland) were grown on Transwells (Corning, New York, USA; 6.5 mm diameter, 0.4 µm pore size). After initiating “air-liquid interface” culture, the basolateral medium (PromoCell, Heidelberg Germany) was replaced with fresh medium and any liquid or mucus on the apical surface was aspirated every 2 days. The apical side was washed prior to infection with 200 µL serum free (SF)-DMEM (no additives). Both the virus stocks and the cell lines were confirmed to be mycoplasma-free using Lookout mycoplasma PCR detection kit. The ALI cultures from each donor were infected with HRSV at a multiplicity of infection (MOI) of 0.1, using SF-DMEM (no additives)-treated cells as mock infection. To calculate the MOI cultures from one well from each donor were trypsinized and enumerated. Just before infection, the apical side was washed twice with 200 µL SF-DMEM (no additives) and fresh medium was added to the basolateral side. Cultures were infected (on the apical side for ALI) by incubation for 2 h at 37 °C 5% CO2 with 200 µL of virus stock diluted in SF-DMEM (no additives) beforehand, or with medium only (mock). Subsequently, the virus inoculum was removed and the apical side was rinsed 4 times with SF-DMEM (no additives). The cultures were kept at 37 °C 5% CO2 and monitored daily by light microscopy for CPE.

### 2.3. Label Free Mass Spectrometry and Informatics

Sample preparation, mass spectrometry, and analysis were adapted from an approach described previously [27]. Soluble proteins were precipitated by the addition of ice-cold 100% (*w*/*v*) trichloroacetic acid (TCA) to a final concentration of 15% (*w*/*v*) with incubation on ice for 2 h. Samples were centrifuged at 14,000× *g* for 10 min to pellet proteins. Pellets were washed three times with ice-cold acetone and were allowed to air dry. Protein pellets were re-solubilised in 50 mM ammonium bicarbonate, 0.1% Rapigest (Waters, Manchester, UK). Protein concentration was determined used the Pierce Coomassie Plus (Bradford) Protein Assay (Fisher Scientific, Loughborough, UK) following the manufacturer’s instructions. Sample protein content and volume was normalized with 50 mM ammonium bicarbonate. Samples were then heated at 80 °C for 10 min, reduced with 3 mM dithiothreitol (Sigma-Aldrich Company Ltd, Gillingham, UK) at 60 °C for 10 min, then alkylated with 9 mM iodoacetimde (Sigma) at room temperature for 30 min in the dark. Proteomic grade trypsin (Sigma) was added at a protein: trypsin ratio of 50:1 and samples incubated at 37 °C overnight. Rapigest was removed by adding TFA to a final concentration of 1% (*v*/*v*) and incubating at 37 °C for 2 h. Peptide samples were centrifuged at 12,000× *g* for 60 min (4 °C) to remove precipitated Rapigest. Samples were desalted using C18 spin tips (Pierce, Fisher Scientific, Loughborough, UK) according to the manufacturer’s instructions.

Peptides were analyzed by on-line nanoflow LC-MS/MS. Peptides were separated using an Ultimate 3000 nano system (Dionex/Thermo Fisher Scientific) equipped with an Easy-Spray PepMap^®^ RSLC analytical column (50 cm × 75 μm inner diameter, C18, 2 μm, 100 Å) fused to a silica nano-electrospray emitter (Dionex, ThermoScientific, Hemel Hempstead, UK). The column was kept at 35 °C. Chromatography was performed with a buffer system consisting of 0.1% formic acid (buffer A) and 80% acetonitrile in 0.1% formic acid (buffer B). The peptides were separated by a linear gradient of 3.8–50% buffer B over 90 min at a flow rate of 300 nL/min. The LC system was coupled to a Q-Exactive mass spectrometer (Thermo Fisher Scientific). The mass spectrometer was operated in data-dependent mode with survey scans acquired at a resolution of 70,000 at *m*/*z* 200. Scan range was 300 to 2000 *m*/*z*. Up to the top 10 most abundant isotope patterns with charge states +2 to +5 from the survey scan were selected with an isolation window of 2.0 Th and fragmented by higher energy collisional dissociation with normalized collision energies of 30. The maximum ion injection times for the survey scan and the MS/MS scans were 250 and 50 ms, respectively, and the ion target value was set to 1E6 for survey scans and 1E5 for the MS/MS scans. MS/MS events were acquired at a resolution of 17,500. Repetitive sequencing of peptides was minimized through dynamic exclusion of the sequenced peptides for 20 s.

Thermo RAW files were imported into Progenesis QI for Proteomics (version 4.1, Nonlinear Dynamics, Newcastle upon Tyne, UK). Peaks were picked by the software using default settings and filtered to include only peaks with a charge state between +2 and +7. Spectral data were converted into .mgf files with Progenesis LC–MS and exported for peptide identification using the Mascot (version 2.3.02, Matrix Science, London, UK) search engine. Tandem MS data were searched against human and HRSV (A and B) databases (Uniprot, Feb 2015, 20,389 sequences; 11,407,585 residues). The search parameters were as follows: precursor mass tolerance was set to 10 ppm and fragment mass tolerance was set as 0.05 Da. Two missed tryptic cleavages were permitted. Carbamidomethylation (cysteine) was set as a fixed modification and oxidation (methionine) set as variable modification. Mascot search results were further validated using the machine learning algorithm Percolator embedded within Mascot. The Mascot decoy database function was utilized and the false discovery rate was < 1%, while individual percolator ion scores >13 indicated identity or extensive homology (*p* < 0.05). Mascot search results were imported into Progenesis LC–MS as XML files. Relative protein abundance was calculated by the Hi-3 default method within Progenesis.

### 2.4. RNA Extraction and Sequencing

Total RNA was extracted from clinical nasopharyngeal aspirate (in triplicate) by use of a Qiagen RNeasy minikit. The RNA concentration was measured by the Qubit RNA BR assay. Agarose gel electrophoresis with ethidium bromide (EtBr) staining was used to visualize RNA in order to check the quality of each RNA sample. cDNA was synthesized from RNA via reverse transcription in order to check the amplification by using an RdRp chain reaction. Samples were quality checked for integrity (Bioanalyzer RNA picochip) and quantity (Qubit RNA kit, Invitrogen Life Technologies Ltd, Paisley, UK). After assessing that the RNA quality was good, with RNA integrity number (RIN) values of 9, the samples were subjected to poly(A) selection by use of a Dynabeads mRNA purification kit (Invitrogen Life Technologies Ltd, Paisley, UK). In this case, 15 μL of beads per sample was washed with binding buffer (2) and resuspended in 30 μL of 2x bead buffer. Samples were made up to 30 μL and heated to 65 °C for 5 min to remove any secondary structure. Samples were mixed with the beads on a rotator for 5 min at room temperature. After collecting the beads on a Magnetic Particle Concentrator (MPC), the beads were washed twice with wash buffer, with removal of all traces after each wash step. Twelve microliters of water were added to the beads for elution, which was achieved by heating the sample at 70 °C for 2 min and retrieving the supernatant containing the RNA. This was assessed for rRNA on an RNA picochip. 

The twice-selected material was all used as input for ScriptSeq assay (Epicentre, Madison, USA). Samples were treated per the manufacturer’s protocol. Samples were mixed with primer and fragmentation buffer, heated at 85 °C for 5 min, and placed on ice. Samples were converted to cDNA and purified with Ampure XP. Samples were mixed with bar-coded primers and amplified with 15 PCR cycles. The libraries were purified with Ampure XP and assessed by use of a Bioanalyzer and an HS DNA kit, and the quantity was determined by use of a Qubit DNA HS kit. Samples were pooled on an equimolar basis for a single lane. 

The quantity and quality of the final pool were assessed using a Qubit kit (Invitrogen Life Technologies Ltd, Paisley, UK) and a Bioanalyzer (Agilent, Cheshire, UK) and then quantitative PCR, using a Kapa library quantification kit on a Roche LC480II Light Cycler machine according to the manufacturer’s instructions. The template DNA was denatured according to the protocol described in the Illumina cBot user guide and loaded at 9 pM. To improve the sequencing quality control, 1% PhiX174 was spiked into the sample. Sequencing was carried out on an Illumina HiSeq 2000 instrument with version 3 chemistry, generating 100-bp paired-end reads. For each time point, the sequence reads were mapped to the HRSV-A2 genome by using Bowtie2. For each alignment to the HRSV genome, the output BAM files were further analyzed using QuasiRecomb [28]. While Bowtie2 aligned more than 1 million reads to the HRSV genome, we selected 1 million alignments at random. We used the coverage option for QuasiRecomb (command line java XX:NewRatio 9 - Xms2G - Xmx10G - jar QuasiRecomb.jar - i reads.sam - coverage) and then parsed the output files with in-house software written in Perl that deter- mined the most abundant nucleotide for each position (i.e., the consensus nucleotide) and reported the frequency of use for the other three nucleotides, as described previously [29,30,31].

## 3. Results

HRSV infection can result in a severe infection in children requiring hospitalization. To investigate the effects of HRSV infection in this patient group, nasopharyngeal aspirates that had been taken for routine diagnostic purposes, were analyzed using quantitative proteomics and RNAseq. These data were also compared to the same analysis on nasopharyngeal aspirates taken from a control group of children, who were negative (at the time of sampling) for HRSV. For the children infected with HRSV, samples were analyzed from children with different viral loads at the time of sampling, and these were then grouped into three arbitrary categories; low viral load (Ct > 25) (*n* = 7), medium viral load (Ct = 20–25) (*n* = 3), and high viral load (Ct < 20) (*n* = 2).

### 3.1. Identification of Viral Proteins in Nasopharyngeal Aspirates

**Figure 1:**
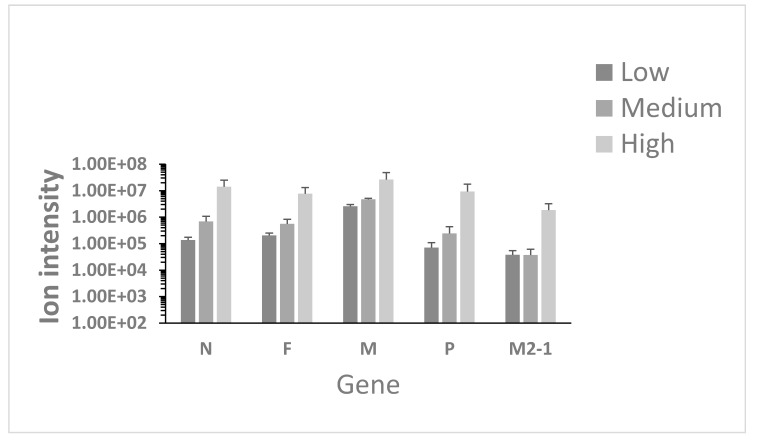
Analysis of summed peptide intensities of Human respiratory syncytial virus (HRSV) proteins identified and quantified in the nasopharyngeal aspirates from children with different viral loads of HRSV, as determined by Ct, low viral load (Ct > 25), medium viral load (Ct = 20–25), and high viral load (Ct < 20).

HRSV and other respiratory pathogens are identified in the clinical setting using a multiplex RT-PCR respiratory pathogen screen panel. Proteomics has been proposed to be used for the potential identification of pathogens in complex samples [32]. However, as with nucleic acid-based approaches, such as sequencing, any search parameters must contain information (e.g., amino acid sequence) of the pathogen being searched for. Conventionally, nucleic acid-based detection systems were thought to be more sensitive than protein-based assays. To evaluate this, LC-MS/MS was used to identify HRSV proteins in the nasopharyngeal aspirate samples. Here, we would predict that viral proteins present in the virion would predominate over viral proteins present intra-cellularly, as nasopharyngeal aspirates should, in the main, represent secretions from the respiratory epithelia. In total five viral proteins were identified and quantified using two peptides or more (Figure 1; N (17 peptides), F (15 peptides), M (14 peptides), P (9 peptides), and M2-1 (5 peptides). In addition, three other viral proteins were identified by a single unique peptide (NS1 and L), but therefore could not be quantified. The peptide abundance profiles also correlated with viral load e.g., viral proteins were more abundant for the highest viral loads, and least abundant for the lowest viral load (See Supplemental Table S1 for ANOVA comparison). Two HRSV proteins were not detected; the NS2 and G proteins, even though that these proteins are encoded by transcripts that have higher predicted abundance than the F, M2-1 and L transcripts, and the G protein is present in virions. The major identified viral proteins are consistent with the presence of virus particles in the nasopharyngeal aspirates rather than proteins from intracellular material, where for example, the NS1 protein has been identified [17].

### 3.2. Identification of Viral RNA in Nasopharyngeal Aspirates

**Figure 2:**
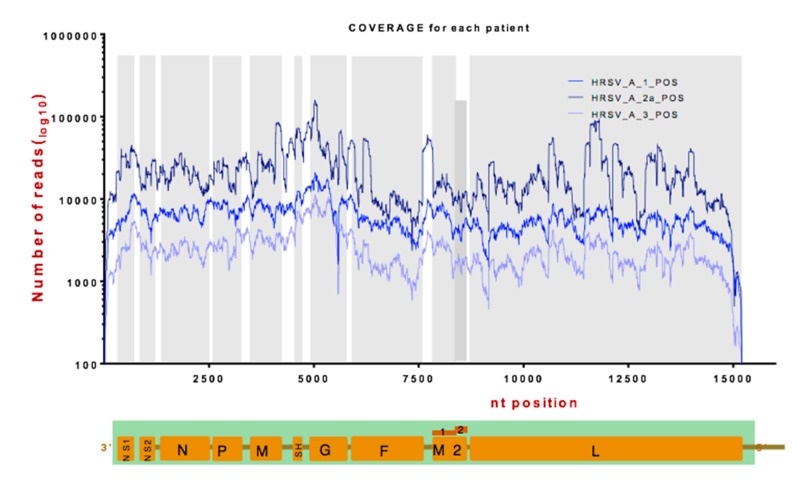
Depth of coverage for each nucleotide of sequence data for HRSV RNA mapped to the HRSV sub-group A2 genome from the three clinical samples (as indicated in the figure). The position of each nucleotide on the HRSV genome is shown on the x-axis with the virus genome and position of each genome as a reference shown below. The number of mapped reads is shown in logarithmic scale.

HRSV has been shown to exhibit genome diversity between patients and isolates, particularly in the G gene [33,34]. Data suggest that the mean rates of nucleotide substitution are approximately 6–8 × 10^−4^ per site per year [35,36]. To investigate the viral genome and minor variants present in the viral genome from the patients, RNAseq was used to sequence the viral RNA present within the nasopharyngeal aspirates. Sufficient read depth for consensus genome and minor variant analysis was obtained from three patients (two with a Ct value of 15, termed A1 and A2a, and one with a Ct value of 20, termed A3) (Figure 2). As would be predicted, the data indicated that more reads mapped to the viral genome in the two samples with the highest viral load compared to the sample with the lower viral load.

**Figure 3:**
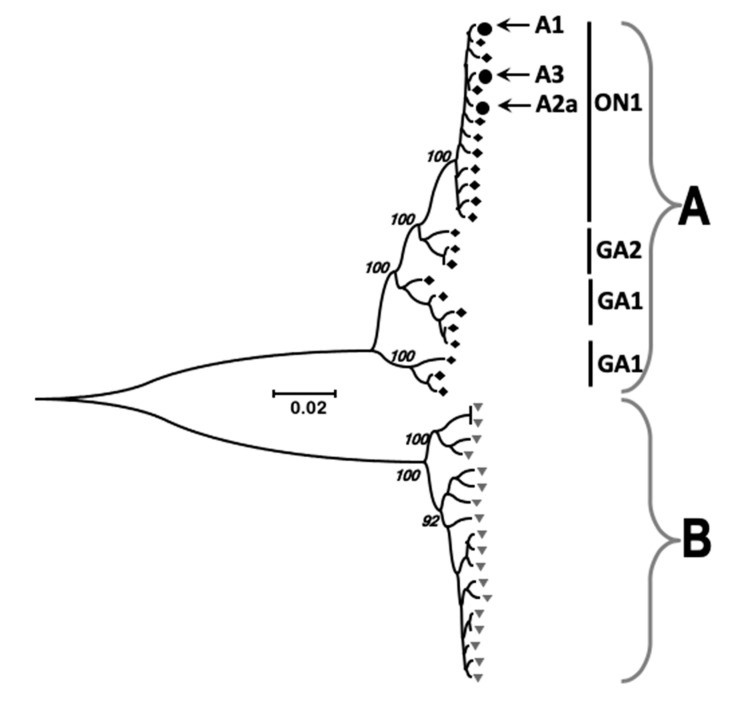
Phylogenetic analysis of the sequence of the three clinical samples compared to examples from sub-group A and sub-group B viruses. The three clinical isolates are indicated with arrows and the group A and B references full genome sequences from the Gene-bank database. Neighbor-joining/Kimura two-parameter phylogenetic analysis was performed using the MEGA7 software package. The data indicated that the three samples sequenced contained HRSV most similar to the ON1 isolate.

Although there were peaks and troughs in reads mapping to the genome, generally the number of mapped reads was equivalent across the genome, apart from the termini (Figure 2). This would be consistent with the nasopharyngeal aspirates containing viral particles rather than intracellular material. In this latter case, we would have predicted to observe a marked decrease in the abundance of the L mRNA, which is the least abundant viral mRNA expressed in cell culture [30]. First, the sequence read depth would have been predicted to follow the transcriptional gradient of viral mRNA synthesis. Second, there was no decrease in reads that were mapped to the intergenic regions compared to the proximal encoded mRNAs. Again, if viral mRNA was present, then reads mapped to the intergenic regions would have been predicted to be underrepresented – which was not the case. The mapped reads were used to assemble a consensus genome for each virus within the patient and also to determine whether minor variants existed. The three assembled consensus genomes were compared with previous HRSV strains and all mapped to subgroup A viruses (Figure 3).

### 3.3. Identification and Comparison of Minor Variants in HRSV in the Clinical Samples Compared to Air Liquid Interface and Cell Culture Systems

Whilst phylogenetics can be used to compare viral genotypes between samples, due to the error prone nature of RNA dependent RNA polymerases, mutations can also occur during virus replication leading to genome diversity within a particularly patient. Various selection pressures (such as the antibody response) can act on the genome with time, leading to the emergence/selection of specific mutations. The RNaseq data can be used to capture information on viral genome variation within a patient, this genome variation can be captured as minor variants and their frequencies [29,31,37]. Any variant that reaches the 50% threshold then becomes part of the consensus genome sequence. By assembling maps of minor variants, we were able to examine the nucleotide variation within the viral genomes obtained from the three clinical samples. For reference this genome variation was compared to an HRSV A2 strain grown in air liquid interface culture (in triplicate) and also using published datasets from our work examining the genome variability of HRSV in cell culture (Hep2 cells) [30]. The air liquid interface (ALI) culture system recapitulates major aspects of the respiratory epithelia [38,39], but in the absence of the adaptive response. The A2 strain was used as this laboratory adapted strain undergoes productive replication in both the Hep2 cells and the ALI culture system. 

**Figure 4:**
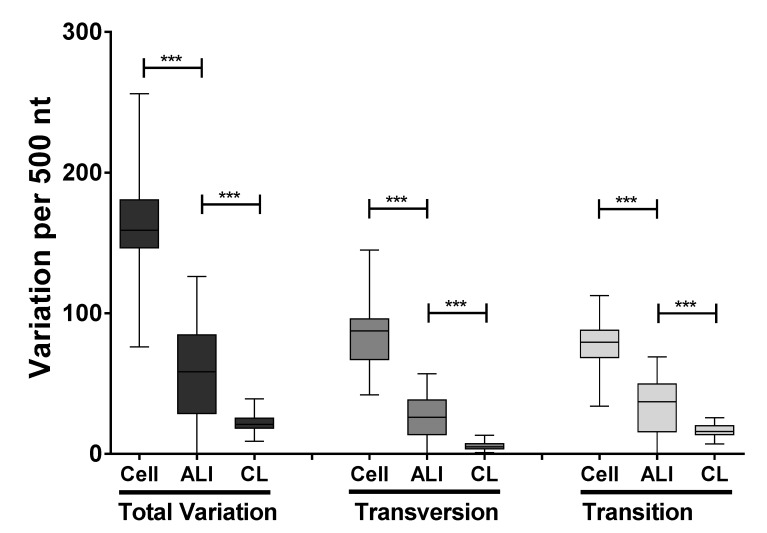
Transition and transversion analyses of each condition. The analysis was implemented using a 500-nucleotide sliding window across the HRSV genome. Each box plot indicates the mean value, the 25th and 75th percentiles, and the 95% confidence interval (CI) of the mean. The analysis was also performed using a 100-nucleotide sliding window and the results were similar. Cell, the air liquid interface (ALI) and clinical samples CL refers to viral RNA sequenced from cell culture, air liquid interface samples and clinical samples. Statistical significance was estimated using Kruskal-Wallis with the significance threshold *p* < 0.05.

**Figure 5:**
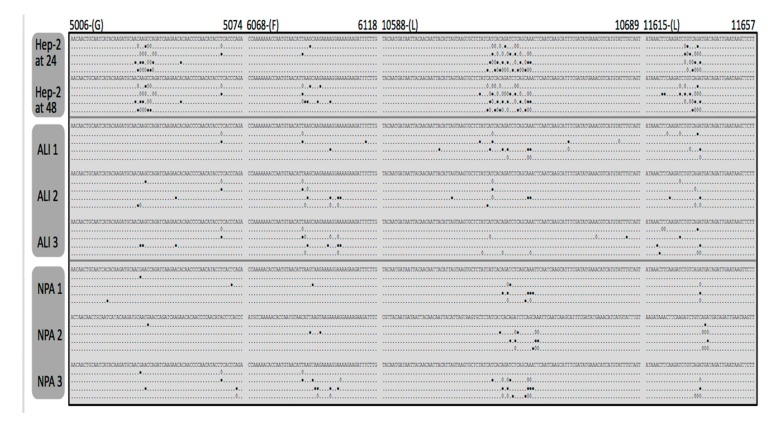
Representative analysis of transitions (full circles) and transversions (open diamonds) resulting from the minor nucleotide variants at exemplar positions of the HRSV genome (as indicated by nucleotide number) for the A2 strain found either in the nasopharyngeal aspirates, the air liquid interface cultures, or the Hep2 cells. For each condition, the consensus sequence (con) is shown on the first line. The following four lines represent each of the four nucleotides found as minor variants. The cut-off value was 0.5%, and the region of the genome shown is indicated by the bold numbers at the top.

The data indicated that clinical sample transition substitutions had a significantly higher frequency than transversion substitutions (*p* < 0.0001) (Figure 4). Overall the total frequency in minor variants observed in the viral genomes sequenced from the clinical samples was less than that from virus grown in either air liquid interface culture or from cell culture (Figure 4). Analysis indicated that the variation did not occur uniformly across the genome and was instead localized to in many cases to discrete clusters (Figure 5). We had previously observed this in virus grown in cell culture and part of the data from the previous study is included for comparison [30]. Interestingly the clusters of variation appeared to be non-random and grouped together for both the virus isolated from nasopharyngeal aspirates, air liquid interface culture, and cell culture (Figure 5). Reflecting the greater nucleotide variation in cell culture derived virus compared to virus derived from air liquid interface or the nasopharyngeal aspirates (Figure 4), there appeared to be greater frequency of variation at these clusters for the cell culture derived virus, compared to either the virus found in the ALI or nasopharyngeal aspirates from the clinic. This would indicate that the ALI may recapitulate viral replication/selection observed in vivo. These hotspots of transition and transversion substitutions appear across the genome and examples of occurrence are shown for the G, F, and L genes (Figure 5).

**Figure 6:**
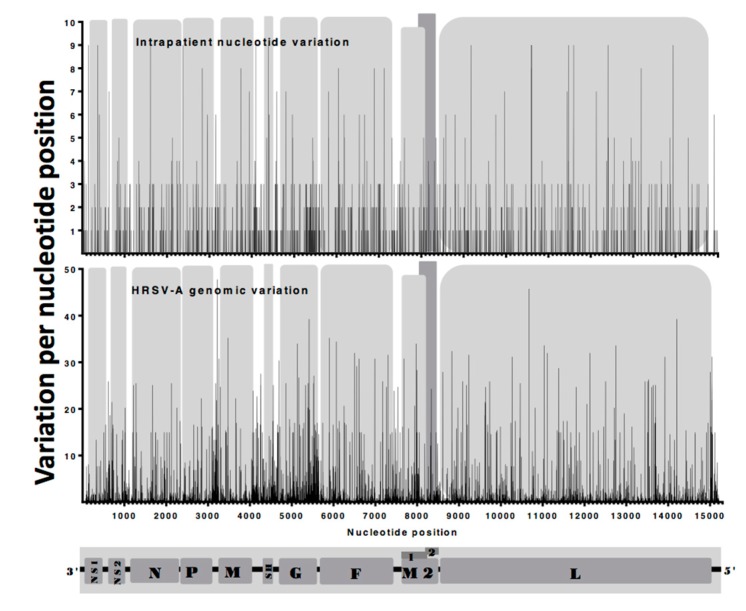
Intra- and inter-patient nucleotide variation. Intra patient variation across the length of the viral genome. The upper panel depicts the normalized intra-patient variation illustrating the number of minor variants at each nucleotide position plotted against the corresponding nucleotide position number. The lower panel depicts the inter-patient variation from a calculated consensus sequences estimated using 241 group A full genome sequences from the Gene-bank database.

Sequence data has demonstrated variability in the G gene sequence between different HRSV strains and a highly variable region has been identified in the gene/protein, and this may result in the antigenic variability of the virus [33,34,40]. Whilst this variability has been identified between different virus isolates, whether this nucleotide variability occurs within a patient is unknown. By assembling minor variants and consensus genomes, we were able to examine both within and between patient variation in the nucleotide sequence (Figure 6). Here the data indicated differences in the minor variants between patients and between viral genomes within the same nasopharyngeal aspirate/patient. Again, distinct clusters of variants along the genome were observed – particularly in the glycoprotein coding region, suggesting underlying variability in this region even within the same patient.

### 3.4. Comparison of the Host Response Between Paediatric Cases with HRSV and Control Cases

Numerous studies have suggested that sequalae in infection with HRSV is associated with the initial innate immune response [18,19] and data to support this has been obtained from in vivo samples [38], in vivo models [41], ex vivo samples [10,22], and cell culture [10]. HRSV encodes several proteins, including the NS1 and NS2 proteins that can associate with host cell proteins and modulate the innate response and other host pathways [21]. To investigate the host response in the clinical nasopharyngeal aspirate samples, mass spectrometry followed by Progenesis analysis was used to identify and quantify cellular proteins. This data was visualized in the form of a heat map. This clustered the changes in abundance of cellular proteins according to whether the samples came from uninfected or infected children. Further, samples from infected children were then classified into categories of viral load; ‘low’ infection (ct score >25), medium (ct score 20–25), and high (ct score <20). Therefore, the analysis indicated that the proteome of the nasopharyngeal aspirates was different between the different infected groups and the control group. This indicated that defined cellular proteins either increased or decreased in abundance in the nasopharyngeal aspirates from infected children compared to control (non-HRSV) children and this was also dependent on viral load. The proteins that were greater in abundance in the proteomes of the nasopharyngeal aspirates sampled from infected children compared to control children included examples of proteins encoded by interferon stimulated genes (e.g., ISG15, ICAM1, IFIT1, and OAS2). Of particular interest were the mucins (e.g., MUCB, MUC5A, and MUC2) and BPI proteins (BPIB6, BPIB1, BPIB3, and BPIB4). Members of these latter proteins have been implicated in the anti-viral response for influenza virus [42] and have been shown to be characteristic of inflammatory lung disease.

## 4. Discussion

HRSV infection causes a mild disease in most children but can cause a severe disease in some children and requires hospitalization, particularly in children with decreased lung function [43] or with underlying health complications. Much of the disease associated with HRSV infection is proposed to be host mediated, where the highest viral load is often before the time of maximum illness [44]. Studying HRSV infection in the lung is complicated by the invasive sampling procedure required to access this organ, particularly in children, where informed consent cannot be directly obtained from the child. Instead proxy tools such as air liquid interface or sampling secretions from the lung provide the next best solution. In the latter case, either nasal swab or nasopharyngeal aspirates will provide indications in the form of cellular proteins that may be associated with infection [16]. The difference between a nasal swab and nasopharyngeal aspirate is that a nasal swab is obtained by inserting a tip of a bulb into the nostril and a nasopharyngeal aspirate requires a suction device and samples more deeply. In children with HRSV the nasopharyngeal aspirate was more useful in detecting HRSV than the nasal swab [45] and was used in the study herein to evaluate HRSV viral load and study the host response. This was achieved through a combination of high-resolution sequencing and proteomics.

Identification of pathogens in samples from patients has relied on techniques such as microscopy, antigens/antibodies or nucleic acid-based approaches such as PCR/RT-PCR. Recently mass spectrometry based approaches have been proposed as disruptive technologies to identify pathogens [46] and have been used to identify bacteria in community deaths from Ebola virus disease [47]. In this study LC-MS/MS identified peptides of HRSV proteins in nasopharyngeal aspirate from children (Figure 1) with confirmed HRSV infection using nucleic acid-based approaches. Only two HRSV proteins, NS2 and G, were not identified by LC-MS/MS, even though these would be predicted to be higher abundance than proteins encoded by downstream transcripts. Particularly for the G protein, this has been observed in previous studies, and may indicate that the G protein/peptides are hard to identify by LC-MS/MS or indicative of protein stability [17,48]. Nevertheless, in general, our data support the proposal that MS based approaches can be used to identify pathogens in biologically relevant material. For example, matrix assisted laser desorption/ionization time of flight mass spectrometry (MALDI-TOF MS) has been used to identify bacterial [47] and viral pathogens in clinical samples [46,49].

RNAseq analysis of the nasopharyngeal aspirates also identified HRSV nucleic through de novo assembly. Phylogenetic analysis demonstrated that the virus present in the nasopharyngeal aspirates most closely mapped to sub-group A virus. Metagenomic analysis indicated that nucleic acid from other viruses did not reach threshold levels of significance and therefore confirmed the findings of the respiratory array. The RNAseq analysis of minor variants in the HRSV genome indicated that these variants occurred at discrete clusters along the genome (Figure 5). Previous work has shown that ribavirin, a therapeutic treatment for HRSV, can induce hypermutation along the genome (30) leading to error catastrophe, and concomitant reduction in viral load. The RNAseq data indicated that whilst minor variants occurred in clusters along the genome, these were increased in frequency in a cell culture model of infection, compared to the more authentic air-liquid interface culture system and from the nasopharyngeal aspirates themselves (Figure 5). This may be due to a greater selection pressure in the ex vivo and in vivo systems compared to the cell culture grown virus. The clustering of these minor variants may have implications for providing ‘noise’ for selection to work upon and also may result in aberrant viral proteins present at a minor level. Nevertheless, this observation suggests that the use of continuous cell culture to study virus evolution may not be entirely the best model. Related to this, was the observation of a large degree of variability in the G gene of HRSV. The G protein is involved in receptor recognition and is one of the major antigenic determinants and for these reasons has been proposed to exhibit a high degree of antigenic diversity for the generation of antibody escape mutants [33]. Our data suggest this mechanism is operating within patients during infection.

**Figure 7:**
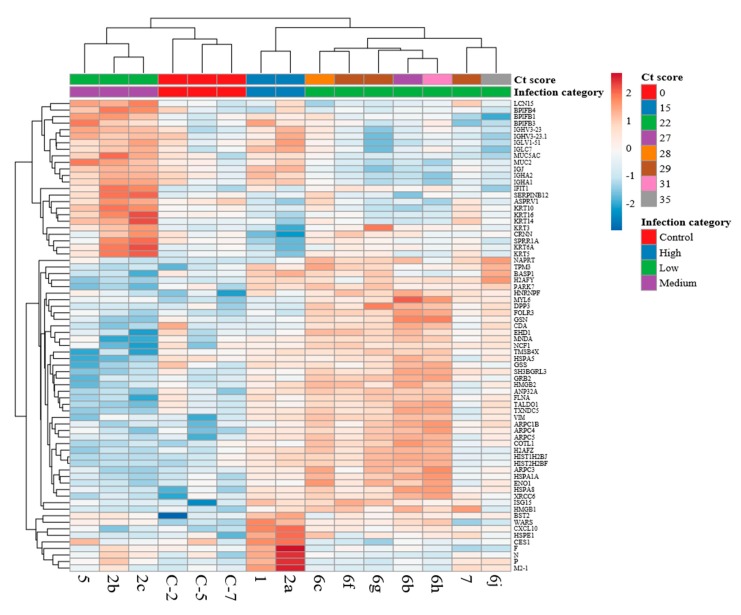
Heatmap of nasopharyngeal aspirate proteomes. The protein (≥ 2peptides, ANOVA *p* < 0.01) abundance profiles obtained with label-free proteomics analysis were clustered in a heatmap using ClustVis online tool. Genes (rows) are clustered are listed on the left edge of the heatmap and samples (columns) clustered on the top edge. Samples were categorized into low, medium, and high infections by viral Ct score (see text). Sample names are highlighted on the bottom edge. Original values are ln(x + 1)-transformed. Rows are centered; unit variance scaling is applied to rows. Both rows and columns are clustered using Euclidean distance and average linkage. 72 rows, 15 columns.

The quantitative proteomic analysis also identified cellular proteins in the nasopharyngeal aspirates and the abundance of some of these proteins changed in abundance in samples taken from children infected with HRSV compared to HRSV-negative children (Figure 7). These increases in protein abundance, in some examples, was linked to viral load, unsurprisingly suggesting that the host response varies according to the amount of virus. Of interest was the identification of several proteins that increased in abundance in HRSV-infected children, belonging to mucins and BPI-containing proteins, the latter having anti-viral properties [42]. For example, depletion of BPIB3 has been shown to enhance coxsackievirus B (CVB) replication [50]. BPI l levels have also been associated with acute pneumonia in adults [51]. Proteomic analysis showed that several cellular proteins, such as HSP70 and HSP90, were associated with HRSV virus particles, suggesting functional relevance and potential therapeutic targets [52]. Indeed, inhibition of HSP70/HSP90 with small molecules had an adverse effect on HRSV biology [53]. Proteomics analysis of bronchoalveolar lavage from calves infected with bovine RSV suggested the activation of neutrophils and that anti-oxidant treatments should be considered to mitigate the physiological effects of infection [54].

Overall, the proteomic characterization of the nasopharyngeal aspirates from HRSV-infected children may identify other potential anti-viral molecules and proteins involved in controlling secretory pathway trafficking. These may provide targets for therapeutic intervention or potential biomarkers for point of care diagnostics to assess the severity of infection and guide clinical interventions.

## Supplementary Materials

The following are available online at https://www.mdpi.com/1999-4915/11/10/926/s1, Table S1: ANOVA Comparison of abundance.
